# The relationship between household chaos and child, parent, and family outcomes: a systematic scoping review

**DOI:** 10.1186/s12889-020-08587-8

**Published:** 2020-04-22

**Authors:** Samantha Marsh, Rosie Dobson, Ralph Maddison

**Affiliations:** National Institute for Health Innovation, School of Population Health, University of Auckland, Auckland, New Zealand

**Keywords:** Household chaos, Scoping review, Family, Child development, Adolescence

## Abstract

**Background:**

Household chaos, represented by the level of disorganisation or environmental confusion in the home, has been associated with a range of adverse child and family outcomes. This review aims to (1) identify how household chaos is measured, (2) chart study details of household chaos literature, and (3) map the existing literature with respect to the relationship between household chaos and child, parent, and family outcomes. We expect that this review will highlight the need to consider the importance of household chaos in child well-being research, particularly in those families where children may be more vulnerable to the adverse effects of household chaos.

**Methods:**

We searched five electronic databases (last updated September 1st 2018) in addition to Google Scholar, and identified publications via a 3-stage screening process, which was conducted by two researchers. Published studies were included if they investigated the association between household chaos and child, parent, or family outcomes. Research that investigated household chaos as a mediator or moderator, or that investigated how the relationship between household chaos and the outcome of interest was mediated or moderated, were also included.

**Results:**

One hundred twelve studies in 111 publications were included. The majority were conducted in the United States (*n* = 71), and used either cross-sectional (*n* = 60) or longitudinal (*n* = 49) study designs. Outcomes of interest were categorised into seven categories: (1) cognitive and academic (*n* = 16), (2) socio-emotional and behavioural (*n* = 60), (3) communication (*n* = 6), (4) parenting, family, and household functioning (*n* = 21), (5) parent outcomes (*n* = 6), (6) hormone (*n* = 8), and (7) physical health and health behaviours (*n* = 19). There was consistent evidence for significant correlations between household chaos and adverse outcomes across all seven categories in diverse populations with respect to age, disease status, and socio-economic status (SES).

**Conclusion:**

There is consistent evidence for associations between household chaos and a number of adverse child, parent, and family-level outcomes. Household chaos may also help describe variations in outcomes between low SES and child development.

Household chaos represents the level of disorganisation or environmental confusion in the family home, and is characterised by high levels of background stimulation, lack of family routines, absence of predictability and structure in daily activities, and an overly fast pace of family life [1, 2]. Importantly, the construct of household chaos has been associated with a diverse range of adverse childhood outcomes, including poorer social-emotional functioning, cognitive development, academic achievement, and behavioural problems [3–9].

Household chaos has been linked with caregiver education, family income, and, perhaps not surprisingly, the number of people living in the household, whereby a lower level of caregiver education, lower family income, and a greater number of people in the home are associated with greater levels of chaos [10]. Despite this, the construct also been demonstrated to be distributed across socioeconomic status (SES) [10], and further, associations between household chaos and adverse child outcomes remain after controlling for SES [3, 11]. For example, one study showed that household chaos was associated with reduced cognitive ability and IQ in children, even after controlling for parent education/IQ, the home literacy environment, parental negativity, parental warmth, stressful events, and housing conditions [3]. Household chaos may therefore represent a unique risk factor for various adverse childhood outcomes, rather than simply reflecting residual confounding with, for example, SES [10].

In addition to the main effects of household chaos, the construct has also been shown to both mediate and moderate relationships between known child risk factors and adverse outcomes. For example, one study documented that the relationship between household chaos and maternal executive function was moderated by SES, suggesting that the adverse effects of household chaos may be exacerbated in socioeconomically distressed contexts [12]. Other studies have also shown that household chaos may mediate relationships between child behavioural problems and bedtime resistance [13], and poverty and socioemotional adjustment [14].

Given the varied ways in which household chaos is associated with adverse child outcomes, it is not surprising that there appears to be growing interest in the construct. Yet despite this interest, and a seemingly large body of evidence demonstrating links between household chaos and a range of adverse child outcomes, no review has been conducted in this field to date. To this end, the goal of this study was to undertake a review to investigate the relationship between household chaos and child-, parent-, and family-level outcomes.

We decided that the ideal method of synthesising the knowledge base at this time, due to the disparate nature of outcomes assessed, age range and disease status of participants, frequency and duration of follow-up, and study designs used, was a systematic scoping review. The scoping review methodology allowed us to (1) investigate how household chaos is measured, (2) summarise the research on how household chaos is included as a primary risk factor of child, parent, and family outcomes, and (3) map the existing literature, with respect to relationships between household chaos and child, parent, and family outcomes. This enabled us to assess not only how household chaos is measured, which is necessary to ensure findings are generalisable across studies, but also what dimensions have been investigated. It also enabled us to summarise the extant scientific research without focussing on a specific outcome, research design, study population (e.g. disease population), or setting [15], therefore allowing us to make recommendations for future systematic reviews and meta-analysis within the field. This review seems timely given that there is also a need to better understand if effects are independent of other known risk factors, or instead reflect an important confounding factor.

## Methods

### Identification of studies

Published scoping review guidelines directed the conduct of this review [16–20]. The protocol for this scoping review was not registered. MEDLINE, PubMed, Embase, PsycINFO, and Child Development & Adolescent Studies were searched from inception through to 1 June 2017, with updated searches run on 8 November 2017 and 1 September 2018. We also ran a general search of ‘household chaos’ in Google Scholar and assessed hits from the first 100 pages of results for eligibility. An iterative process was used to develop the search strategy. Words associated with potential child, parent, and family outcomes (e.g. attention, aggression, diet, sleep, literacy, parent-child interactions) were combined with words and concepts associated with household chaos (e.g. household chaos, family disorganisation), and words associated with our sample of interest (e.g. children, adolescents, mothers, fathers, family). These combinations were used to form search strings, which were applied and run in the different databases. Filters were used to limit the search to studies available in English. The reference lists of all included manuscripts were searched for any additional articles not identified in by the electronic search. Full details of the MEDLINE search strategy are provided in Table 1. The grey literature was not searched.

**Table 1:** Search strategy utilised for MEDLINE (from inception to September 2018)

Search	Search Term	Combination
1	Infant/ or Child/ or Adolescent/ or Mothers/ or Fathers/ or Parents/ or Family/ or child.mp. or infant.mp. or mother.mp. or father.mp. or parent.mp. or adolescent.mp. or teenager.mp. or children.mp.	
2	(Family disorganization or Family disorganisation or hurried).mp. or (confusion adj2 hubbub).tw. or household disorganisation.mp. or household disorganization.mp. or (environmental chaos adj5 family).mp. or (environmental chaos adj5 home).mp. or (environmental chaos adj5 household).mp. or (environmental chaos adj5 house).mp. or (chaotic environment adj5 family).mp. or (chaotic environment adj5 home).mp. or (chaotic environment adj5 household).mp. or (chaotic environment adj5 house).mp. or (chaos adj5 family).mp. or (chaos adj5 home).mp. or (chaos adj5 household).mp. or (chaos adj5 house).mp. or (chaotic adj5 family).mp. or (chaotic adj5 home).mp. or (chaotic adj5 household).mp. or (chaotic adj5 house).mp.	
3	(Child health or Obesity or Overweight or Sleep or Diet or nutrition or “screen use” or Television or “family meals” or eating or Self-Control or self-regulation or Anxiety or Stress or “effortful control” or Attention or aggression or Decision Making or Resilience or family functioning or Parenting or Family Conflict or parental attitudes or Parenting or academic achievement or reading or literacy or mathematics or language or cognition or socio-emotional or social-emotional).mp.	
4		1 and 2 and 3
5		Limit 4 to (english language and humans)

### Study selection

Publications identified through our search strategy went through three stages of screening. During the first stage, titles were screened to establish their relevance to the research question. If relevance could not be established from the title, the study was included in the next stage of screening. During the second stage, abstracts of the selected titles were reviewed according to our inclusion criteria. During the third step, full text articles of the abstracts selected during the previous stage were retrieved and further assessed for relevance. Two researchers conducted this process and disagreements were settled first by discussion and, if necessary, by consultation with a third researcher.

### Inclusion criteria

We included any published research that investigated the association between measures of household chaos and child, parent, or family outcomes, including health, cognitive, and psychosocial outcomes, as well as family-level processes. We also included any published research that investigated household chaos as a mediator or moderator and any studies that investigated how the relationship between household chaos and the outcomes of interest were mediated or moderated.

Due to the scoping nature of this review, we included healthy children and parents, in addition to those with diagnosed medical conditions, developmental delays, and behavioural disorders. No limits were set on study design. We included any study that investigated the effects of the household chaos construct; including studies that used terms such as home chaos, environment chaos (restricted to the home), chaotic homes/households, hurried homes, family chaos, chaotic families, and chaotic living. In addition, given that household disorganisation is a defining feature of household chaos, we also included studies that investigated the effects of family disorganisation and household instability.

While lack of family routines and family meals may represent one dimension of the household chaos construct, alone they are not a proxy measure for household chaos. Recently it was shown that household chaos and family routines may actually represent two distinct constructs and, further, that lack of family routines represents a pathway through which household chaos adversely affects child outcomes [21]. For these reasons, studies focusing primarily on routines (and those specifically using the Family Routine Inventory) and/or frequency of family meals only were not included in the review.

### Data extraction and synthesis

For each included trial, two authors extracted data using an extraction form designed and pre-tested for the purpose of this review. Final data extraction was completed in October 2018. Information extracted from each eligible study included: author(s), year of publication, manuscript title, name of cohort/study, study location, health status of child and/or parent, diagnosed conditions of child and/or parent, SES details, location details (i.e. rural or urban), study aim, whether household chaos was the primary focus of the study, study design, duration and number of follow-ups, whether the study investigated twins/parent-child dyads/individual child/family, number of children/families included, age range of included participants, ethnicity, how household chaos was measured, whether household chaos was reported in the results, outcomes of interest (specifically related to household chaos), whether household chaos was assessed as a mediator and/or moderator, whether the relationship between household chaos and the outcome was mediated and/or moderated, and study results.

Data were summarised to record the number of studies retrieved by country, year of publication, study type, and any characteristics of the population (e.g. low SES, disease status). Outcomes of interest were categorised into seven broad categories (Table 2): (1) cognitive and academic (2) socio-emotional and behavioural, (3) communication, (4) parenting, family, and household functioning, (5) parent outcomes (6) hormone, and (7) physical health and health behaviours. Studies could be included in multiple categories of outcomes if they reported outcomes that fell across different categories. A narrative review is provided for each outcome measure of interest.

**Table 2:** Specific outcomes by outcome category

Outcome Category	Specific Outcomes Assessed
Cognitive and academic	Academic achievement/performance Cognitive ability/outcomes/development Executive function IQ Study skills Reading comprehension Intellectual functioning
Socio-emotional and behavioural	*Response to challenge and task persistence:* • Response to academic challenge • Task persistence • Processing speed *Behavioural and social:* • Behavioural (including peer problems, conduct problems, impulsivity, aggression, anger, and prosocial behaviour) • Anxiety and stress • Social-emotional (including social problems, social competence, adjustment issues, and negative emotionality) • Attention/ADHD • Risk taking (including substance use, sexual activity, violence-related risk-taking, delinquency, and cheating) • Self-regulation and inhibitory control • Depression • Temperament • Future beliefs and aspirations • Empathy • Callous-unemotional traits and psychopathic characteristics
Communication	Verbal/expressive vocabulary and non-verbal abilities Receptive vocabulary
Parenting, family, and household functioning	*Parenting:* • Differential parenting • Parent-child closeness • Parenting – general • Parental self-efficacy • Parental mood • Harsh parenting • Discipline • Sibling relationships • Sleep-disturbing behaviours of family members • Parental reactions • Maternal attribution bias • Emotional availability • Child representation of family dysfunction *Household characteristics and food security:* • Food security • Family meal atmosphere
Parent outcomes	Maternal executive function Parent diet/eating behaviours/weight status Maternal depression Maternal fatigue Maternal/parent sleep
Hormone	Cortisol levels/profile Autonomic nervous system activity Developmental stability of hypothalamic-pituitary-adrenal axis activity Gut biomarkers Inflammatory profile
Physical health, health behaviours, and communication disorders	*Health/disease/disorder outcomes:* • Child weight/weight status • HbA1c • Child health • Stuttering severity • Physical health symptoms *Diet and Dietary behaviours:* • Eating in the absence of hunger • Maternal feeding goals • Food-related behaviours *Sleep:* • Sleep • Sleep anxiety *Other health behaviours/outcomes:* • TV viewing behaviours in a laboratory setting • Maternal perceptions about physical activity

## Results

**Fig. 1:**
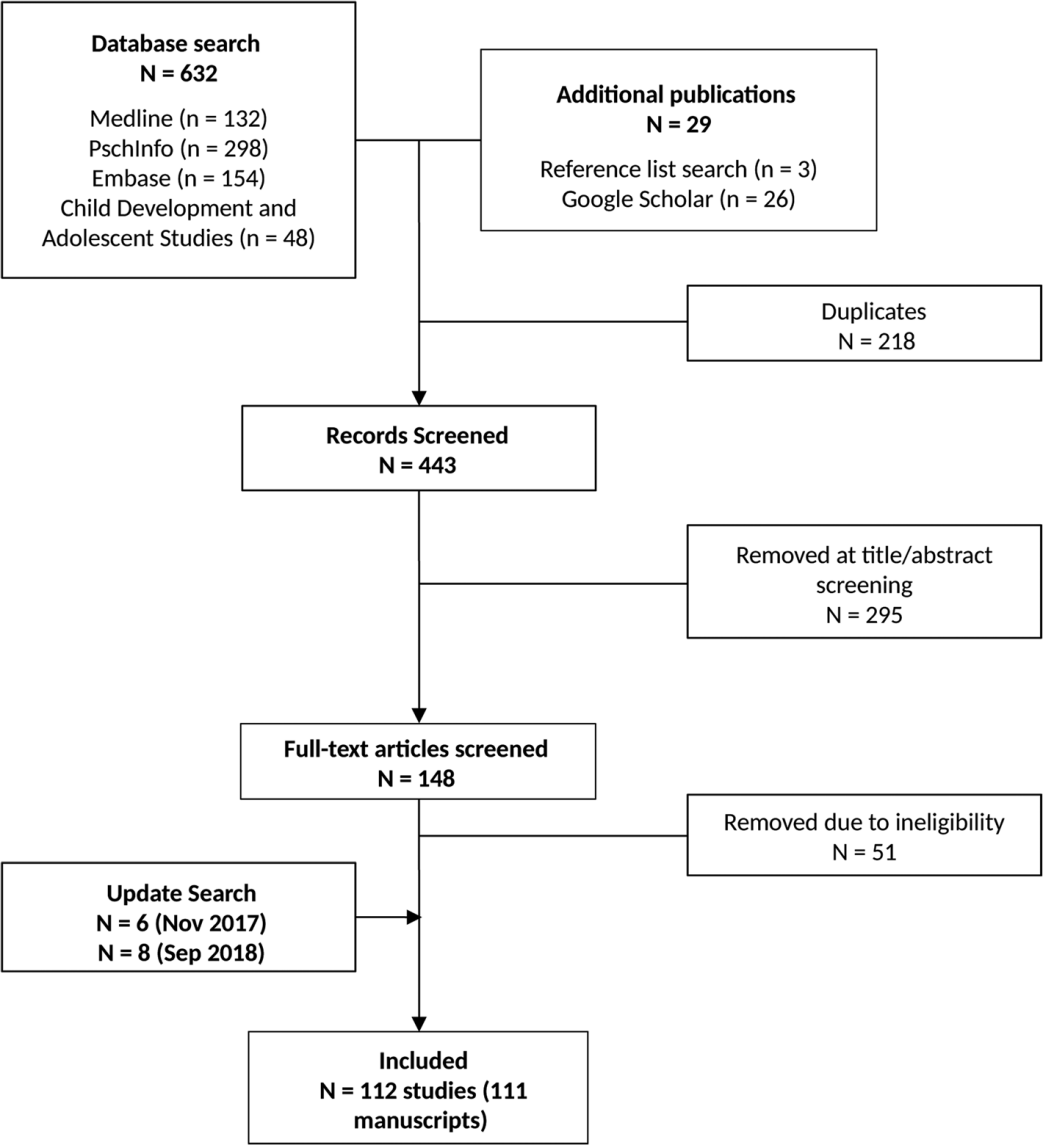
Illustrates the different steps of the data collection process

A total of 661 manuscripts were identified from searches of databases, Google Scholar, and reference lists, of which 218 were duplicates, leaving 443 that were screened for eligibility. Of these, 295 were removed at the title and abstract screening stages, leaving 148 for eligibility screening of full-text articles. After removal of 51 articles due to ineligibility, and inclusion of an additional 6 and 8 articles from the two updated searches in November 2017 and September 2018, respectively, 111 manuscripts (representing 112 studies) were considered eligible and included in the review (Fig. 1).

Studies were excluded for a number of reasons: (1) household chaos was included within a composite measure of family functioning or household environment, (2) no child, parent, or family outcomes were reported, (3) only predictors of household chaos were assessed, (4) the construct of household chaos was not adequately measured (i.e. a different construct was measured), (5) household chaos was only included as a covariate in the analysis and not reported in the results, (6) the paper reported simulation data, and (7) the paper was retracted.

A summary of characteristics of the included studies can be found in Table 3. Overall there was generally an even mix of cross-sectional and longitudinal study designs (*n* = 57 vs *n* = 52), in addition to two experimental/laboratory studies and one case-control study. With respect to the longitudinal studies, samples were primarily drawn from large, nationally representative, longitudinal cohorts, which was reflected in the relatively large number of analyses conducted in sample sizes of greater than 1000 participants (24/104; 23%).

**Table 3:** Summary of characteristics of included studies

Study Characteristic		N^**a**^
Type of study	Cross-sectional	60
	Longitudinal	49
	Experimental/laboratory	2
	Case-control	1
Sample Size	< 100	16
	100- < 1000	72
	1000- < 10,000	22
	≥10,000	2
Cohort/Study	TEDS	12
	FLP	9
	Head Start Cohort	6
	MCS +/− NESS	5
	Pakistan cohort	5
	Western Reserve Reading Project	4
	Stand-alone Head Start Cohorts	4
	Florida Twin Project OR from the Florida State Twin registry	2
	SECCYD	2
	SIBS	2
	Early Alliance Trial	1
	NAPLAN	1
	BIDS	1
	Early Growth and Development Study	1
	ESM	1
	FACES, 2006	1
	Food and Family Project	1
	Fragile Families and Child Well-being Study	1
	Guelph Family Health Study	1
	Home Environment Comparison Study	1
	PHDCN	1
	Social and Educational Change (RISE) study	1
	Wisconsin Twin Project	1
	L-CID	1
	SIESTA	1
	Social and Character Development Research Program	1
	Stand-alone studies/No study or cohort name	46
Context/Participants	Low income/rural	34
	Twins	19
	Adopted children Child Diagnosis/Risk factor:	1
	ADHD	2
	Autism	1
	Obesity	2
	DRD4 risk	1
	Stuttering	1
	Sickle cell disease	1
	Type 1 diabetes	2
	Parent Diagnosis/Risk factor:	
	Depression/	3
	Bipolar disorder	2
	ADHD symptomology	4
Measure of Household Chaos Construct	CHAOS long form	44
	CHAOS short form	42
	CHAOS adapted	10
	Other – questionnaire and/or direct observation	16

Studies were included multiple times, for example, if the study was conducted in more than one country, or a manuscript reported results from more than one study. Where data were taken from a longitudinal cohort but analysis was only conducted at one time point, studies were classified as cross-sectional. Where location of study was not reported, location of the first author was used, and where studies investigated participants as parent-child dyads, families, or twin pairs, the unit of participants was used for sample size reporting (e.g. 5000 twin pairs was reported as a sample size of 5000, and 75 parent-child dyads was reported as a sample size of 75)

*TEDS* Twins Early Development Study, *FLP* Family Life Project, *MCS* Millennium Cohort Study, *NESS* National Evaluation of Sure Start Impact Study, *SECCYD* Study of Early Child Care and Youth Development, *SIBS* Sisters and Brothers Study, *NAPLAN* Australian Twin Study of the National Assessment Program - Literacy and Numeracy, *BIDS* Ben-Gurio University Infant Developmental Study, *ESM* Early Steps Multisite project, *FACES 2006* Family and Child Experiences Survey, 2006 cohort, *PHDCN* Project on Human Development in Chicago Neighbourhoods, *L-CID* Leiden Consortium on Individual Development, *SIESTA* Study of Infant’s Emergent Sleep TrAjectories, *ADHD* Attention-deficit/hyperactivity disorder, *DRD4* Dopamine Receptor D_4_

^a^*N* = 112 studies reported in 111 manuscripts

**Fig. 2:**
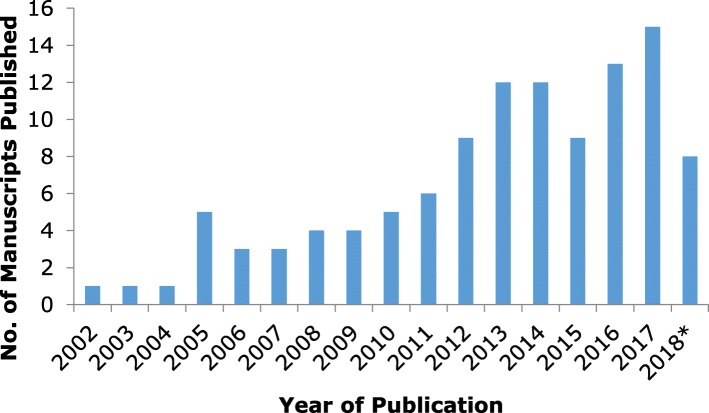
Number of household chaos publications by year

**Fig. 3:**
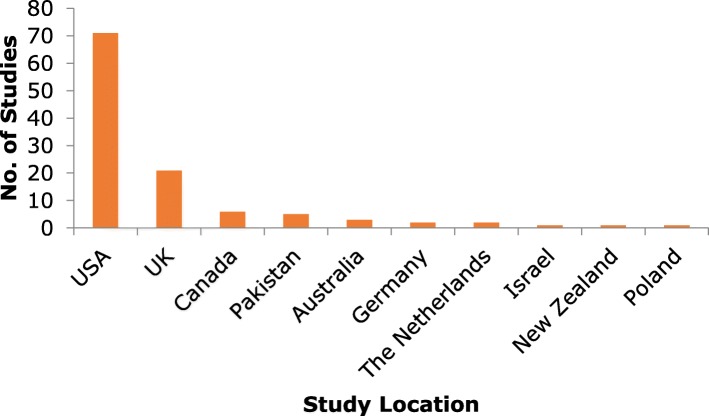
Number of published studies/analyses by country

There has been an increasing trend in recent years in the number of manuscripts investigating the relationship between household chaos and child/parent/family outcomes (Fig. 2), with the majority of publications from research conducted in the USA (*n* = 74) and the UK (*n* = 21) (Fig. 3).

### Methods of household chaos assessment

Studies varied somewhat in the method of household chaos assessment, although overwhelmingly the most frequently used methods were the long-form version of the Confusion, Hubbub, and Order Scale (CHAOS) [22] (*n* = 44) and the short-form version of CHAOS [23] (*n* = 42). The long-form version consists of 15 questions in a True-False response format, with each item reflecting household characteristics that directly represent a chaotic home environment, while at the same time specifically excluding any items that represent adequacy of the dwelling or quality of furnishings, or imply unsanitary conditions. The questionnaire was developed to be used in families regardless of the child’s age, and has been demonstrated to have satisfactory internal consistency, test-rest reliability, and adequate psychometric properties [22]. The short-form version of CHAOS consists of six items rated on a five-point scale (1 = definitely untrue, 5 = definitely true), which has been demonstrated to have acceptable internal consistency [23]. Another 10 studies used adapted long- and short-versions of CHAOS, and the remaining 16 studies relied on questionnaires developed specifically for the study and/or direct observation in the home.

### Outcomes of interest

A summary of study characteristics, including sample size, age of participants, study design, SES status of participants, measure of household chaos, and outcome/s of interest can be found in Table 4. Here we provide a brief narrative review of study findings, with outcomes of interest mapped into seven categories.

**Table 4:** Outcomes of interest

Outcome of interest	N	Twin	Age^**a**^	Design^b^		Low ses	Measure of HC	Outcomes Assessed
				C/O	L			
**Cognitive & Academic** [3, 7, 11, 24–36]								
Asbury, 2006 [24]	2017	✓	0 m, 4y, 7y		✓		CHAOS short form	HC as moderator between non-shared environment variables and academic achievement
Berry, 2016 [25]	1235		2 m, 7 m, 2y, 3y, 5y		✓	✓	Interview/ observation	Academic achievement; executive function
Deater-Deckard, 2009 [3]	302	✓	4-7y	✓	✓		CHAOS short form	IQ
Garrett-Peters, 2016 [26]	1236		6, 15, 24, 36, 48, 58 m & kindergarten (USA)		✓	✓	Interview/ observation	HC as mediator between family income poverty and academic achievement in kindergarten
Hanscombe, 2011 [27]	2337	✓	9 & 12y		✓		CHAOS long form	Academic performance
Hart, 2007 [11]	287	✓	Kindergarten or 1st grade, then 1st or 2nd grade (USA)		✓		CHAOS short form	General cognitive ability; HC as mediator between shared environmental variance and cognitive ability
Hur, 2015 [28]	444		36-70 m	✓			CHAOS short form	HC as mediator between parent depressive symptoms and child cognitive outcomes
Johnson, 2008 [7]	455	✓	mean 6.1 years				CHAOS short form	Woodcock score
Pike, 2006 [29]	5765	✓	0-3y, then 4y				CHAOS short form	Cognitive development
Shamama-tus-Sabah, 2010 [30]	203		8-11y	✓			CHAOS long form	Academic achievement
Shamama-tus-Sabah, 2011 [31]	101		8-11y	✓			CHAOS long form	Learning at school
Shamama-tus-Sabah, 2011 [33]	203		8-11y	✓			CHAOS long form	Study skills
Shamama-tus-Sabah, 2011 [32]	203		8-11y	✓			CHAOS long form	Cognitive performance
Taylor, 2014 [35]		✓	7-13y	✓			CHAOS short form	HC as mediator between respect for rules and reading comprehension
Taylor, 2017 [34]	269		9-16y	✓			CHAOS short form	Reading comprehension
Yarboi, 2017 [36]	65		6-16y	✓			Interview/ observation	Intellectual functioning; academic achievement; executive functioning
**Socio-emotional & Behaviorual** [3, 4, 6, 10, 14, 24, 25, 28, 29, 31–33, 37–83]								
**Response to Challenge & Task Persistence**								
Brown, 2008 [42]	96		3-5y	✓		✓	Interview/ observation	Response to academic challenge
Fuller-Rowell, 2015 [50]	256		9.2, 13.1, 17.5y		✓		CHAOS adapted	HC as a moderator between lower SES and task persistence
Tomalski, 2017 [72]	71		5.5 m	✓			CHAOS long form	Processing speed
**Behavioural & Social**								
Asbury, 2003 [37]	4706	✓	4y	✓			CHAOS short form	HC as a mediator and moderator in the relationship between parenting and behavioural outcomes
Asbury, 2006 [24]	2017	✓	0 m, 4y, 7y		✓		CHAOS short form	HC as moderator between non-shared environment variables and anxiety, hyperactivity, CP, and peer problems
Berry, 2016 [25]	1235		2 m, 7 m, 2y, 3y, 5y		✓	✓	Interview/observation	Social problems
Bobbitt, 2016 [38]	2447		3-4y, 3.5–4.5y (6 m follow-up)		✓	✓	Interview/observation	Behavioural problems; social skills
Bridgett, 2013 [39]	85		4 m	✓		✓	CHAOS long form	HC as mediator in relationship between maternal self-regulation and infant negative emotionality
Brieant, 2017 [40]	167		13-14y		✓		CHAOS short form	HC as moderator in relationship between parental EF and impulsivity and adolescent EF
Brown, 2010 [41]	123		4–7.5y	✓		✓	CHAOS long form	Child attention (assessed by both parent report and computer task)
Calam, 2012 [43]	48		2-10y	✓			CHAOS short form	Child emotional difficulties
Chatterjee, 2015 [44]	929		Mean 16.4y	✓			CHAOS short form	Substance use; sexual activity; and violence-related risk behaviours
Chen, 2014 [45]	149		3-7y	✓			CHAOS short form	Temperament-by-parenting interactions^d^; HC as moderator
Coldwell, 2006 [4]	118		4-8y	✓			CHAOS short form	Child problem behaviour; HC as a moderator in relationship between parenting and child behaviour
Deater-Deckard, 2009 [3]	302	✓	4-7y		✓		CHAOS short form	CP
Dumas, 2005 [10] (study 1)	106		4-6y	✓			CHAOS long form	Behaviour problems; social competence; anger; aggression; attention; ability to understand and respond to social cues
Dumas, 2005 [10] (study 2)	676		7-11y	✓		✓	CHAOS long form	Behaviour problems (caregiver and teacher report)
Evans, 2005 [14]	339		Mean 9.2y at baseline		✓	✓	CHAOS long form + 2 x questions on rituals and routines	HC as mediator in relationship between SES and socio-emotional adjustment
Farbiash, 2014 [46]	84		4y at follow-up		✓		CHAOS short form	HC as mediator in relationship between parental ADHD symptoms and preschool aggression
Fisher, 2018 [80]	390		8y at baseline, 15y at follow-up		✓		CHAOS long form (14 questions)	Psychopathic characteristics; CU; empathy; anxiety; aggression; delinquency; classroom climate quality as moderator of relationship between HC and adolescent outcomes
Flouri, 2015 [48]	10,086		3y, 5y, 7y		✓		3 questions from CHAOS + 2 questions about bedtime and mealtimes	Occupational aspirations at age 7 years
Flouri, 2017 [47]	180		3y, 5y, 7y, 11y		✓		3 x CHAOS questions	HC as mediator in relationship between low SES and CP in children with ADHD
Fontaine, 2011 [49]	9578		4y, 7y, 12y		✓		CHAOS short form	CP; CU traits
Gould, 2017 [51]	520	✓	6-15y		✓		CHAOS short form	HC as moderator of relationship between genes and development of ADHD
Gregory, 2005 [52]	6612		3y, 4y		✓		CHAOS short form	Anxiety; HC as mediator of relationship between sleep problems and anxiety
Gunzenhauser, 2017 [53]	263		Mean 8.59y		✓		CHAOS long form	Self-regulation
Hannigan, 2017 [54]	6646	✓	9y, 12y, 14, 16y		✓		CHAOS short form	Depressive symptoms
Hardaway, 2012 [55]	731		Mean 3.4y		✓	✓	CHAOS long form	Inhibitory control; problem behaviour; aggression
Human, 2016 [56]	261		13-16y at baseline (2-year follow-up)		✓		CHAOS long form	Depressive symptoms; perceived stress
Hur, 2015 [28]	444		36-70 m	✓			CHAOS short form	HC as mediator between parent depressive symptoms and child socio-emotional development, and behavioural self-regulation
Jaffee, 2012 [57]	6286	✓	9y, 12y		✓		CHAOS short form	CP; hyperactivity-inattention
Kahn, 2016 [58]	115		Mean 13.97y	✓			CHAOS short form	Moderated mediation analysis = Harsh parenting as a mediator in the relationship between parent CU traits and child CU traits, and then HC as a moderator of this relationship
Kim-Spoon, 2017 [59]	167		13-14y		✓		CHAOS short form	HC as moderator of relationship between parental control and social competence
Lauharatanahirun, 2018 [81]	167		13–15 years		✓		CHAOS short form	HC as moderator between parental knowledge and insular risk processing
Laurent, 2014 [60]	200		9 m, 18 m, 27 m, 4.5y, 6y		✓		CHAOS short form	Behaviour problems in adopted children
Lemery-Chalfant, 2013 [61]	807	✓	Mean 7.93y	✓			CHAOS short form	Genes as mediator in relationship between HC and child temperament (passive gene-environment); HC as moderator in relationship between genes and child temperament
Martin, 2012 [6]	1266		2.5y, 5y		✓		Questionnaire designed for study	Attention; aggression; delayed gratification; Mediating role of maternal warmth and provision of learning materials in the home on the relationship between lack of routines and delayed gratification
Midouhas, 2013 [62]	209		9 m, 3y, 5y, 7y		✓		3 items from the CHAOS short form	CP in children with autism; HC as moderator of relationship between poverty and parenting, and psycopathology in children with autism
Miller, 2017 [63]	380		Preschool (USA)	✓		✓	CHAOS long form	Negative liability; emotion regulation
Mills-Koonce, 2016 [64]	~ 1292		2 m, 6 m, 15 m, 24 m, 36 m, first grade		✓	✓	Interview/observation	CU; CP; empathy; HC as moderator in relationship between parenting and child behaviours; Maternal sensitivity and harsh parenting as mediators in relationship between HC and child behaviour
Oliver, 2008 [65]	285	✓	8-10y	✓			CHAOS short form	HC as moderator in relationship between within-pair differences in perceptions of the classroom environment and teacher report of less prosocial and more hyperactive behaviour, and greater conduct and peer problems
Panico, 2014 [66]	6572		3y, 5y		✓		3 items from CHAOS	Behavioural outcomes
Pike, 2006 [29]	5765		0-3y, 4y				CHAOS short form	Behavioural outcomes
Raver, 2015 [67]	1025		Followed between 6 m and 58 m		✓	✓	Interview/observation	Ability to recognise and modulate negative emotions
Shamama-tus-Sabah, 2011 [31]	101		8-11y	✓			CHAOS long form	Attention
Shamama-tus-Sabah, 2011 [68]	203		8-11y	✓			CHAOS long form	CP
Shamama-tus-Sabah, 2011 [32]	203		8-11y	✓			CHAOS long form	Internalising and externalising behaviours
Shamama-tus-Sabah, 2011 [33]	101		8-11y	✓			CHAOS long form	Adjustment problems
Shapero, 2013 [69]	956		Followed between birth and 15y		✓		CHAOS long form	Changes in emotional and behavioural problems between childhood and adolescence
Shelleby, 2014 [70]	731		2-3y, 4-5y, 7.5–8.5y		✓	✓	CHAOS long form	HC as mediator in relationship between income and emotional problems and CP
Supplee, 2007 [71]	120		2, 3, 4 & 5y		✓	✓	CHAOS long form	Externalising behaviours; Maternal monitoring as moderator in relationship between HC and externalising behaviours
Towe-Goodman, 2011 [73]	636		Infant, toddler, 3y		✓	✓	CHAOS long form	HC as moderator in relationship between interparental aggression in toddlerhood and the development of child attention skills and early childhood behaviour problems at age 3 years, in low-income families
Tucker, 2015 [74]	325		Mean 15.71y	✓			CHAOS short form	Adolescents’ future beliefs; Mother’s hostility as moderator in relationship between HC and adolescents’ future beliefs
Tucker, 2018 [82]	238		Followed between 10th grade (mean 15.59y) and 12th grade		✓	✓	CHAOS short form	Problematic substance use, depression; HC as moderator in relationship between hostile parenting and depression
Valiente, 2007 [75]	188		7-12y	✓			CHAOS short form	Effortful control; externalising behaviours
Vernon-Feagans, 2016 [76]	1145		2, 6, 15, 24, 48, & 60 months		✓	✓	Interview/observation	Regulatory behaviours in kindergarten; Parenting and executive function as mediators of the relationship between HC and behaviours
Vilsaint, 2013 [77]	364		2y, 5y		✓	✓	CHAOS long form	Internalising & externalising behaviours
Vrijhof, 2018 [83]	201	✓	34-66 m	✓			CHAOS long from	Effortful control; cheating behaviour
Wang, 2012 [78]	304	✓	Mean 6.07y, 7.15y, & 8.30y		✓		CHAOS short form	HC as moderator in the genetic link between attention regulation and externalising behaviours
Wilkinson, 2013 [79]	~ 8800	✓	2, 6, 15, 24, 48, & 60 months		✓		CHAOS short form	HC as moderator in relationship between genes and depressive symptoms (Gene × Environment interaction)
**Communication** [6, 7, 23, 25, 84, 85]								
Asbury, 2005 [84]	2223	✓	4y	✓			CHAOS short form	Verbal and non-verbal abilities; HC as moderator in the heritability of verbal and non-verbal abilities
Berry, 2016 [25]	1235		2 m, 7 m, 2y, 3y, 5y		✓	✓	Interview/observation	Receptive vocab
Johnson, 2008 [7]	455	✓	mean 6.1 years				CHAOS short form	Expressive vocab and phonological awareness
Martin, 2012 [6]	1266		2.5y, 5y		✓		Questions designed for study	Receptive vocab; Mediating role of provision of learning materials in the home on the relationship between lack of routines and receptive vocab
Petrill, 2004 [23]		✓	3 & 4y		✓		CHAOS short form	Verbal and non-verbal abilities; HC as mediator in relationship between shared environment and verbal and non-verbal scores
Vernon-Feagans, 2012 [85]	1145		2, 6, 15, 24, & 36 m		✓	✓	Interview/observation	Receptive and expressive language
**Parenting, Family & Household Functioning** [4, 10, 45, 75, 85–101]								
**Parenting**								
Atzaba-Poria, 2008 [86]	344		4-5y and an older sibling aged <=8y	✓			CHAOS short form	Differential parenting
Barnes, 2014 [87]	24,610		3y	✓			3 questions from CHAOS	Parent/child conflict & closeness; home learning environment; parenting
Chen, 2014 [45]	149		3-7y	✓			CHAOS short form	Temperament-by-parenting interactions^d^; HC as a moderator
Coldwell, 2006 [4]	118		4-8y	✓			CHAOS short form	Parenting
Corapci, 2002 [88]	57		12-14 m	✓			CHAOS long form	Parenting; Parental self-efficacy and parent negative mood as mediators in relationship between HC and parenting
Deater-Deckard, 2012 [89]	147		3-7y	✓			CHAOS short form	HC as a moderator of maternal EF, which itself was assessed as a moderator in the relationship between child CP and harsh parenting
Dumas, 2005 [10] (study 1)	106		4-6y	✓			CHAOS long form	Parental discipline, and accuracy and efficiency in a co-operative parent-child interactional task
Kretschmer, 2009 [91]	118		4-8y	✓			CHAOS short form	Sibling relationships; Parenting as a mediator and moderator in the relationship between HC and sibling relationships
Mokrova, 2010 [92]	319		7y (group 1) & 10y (group 2)	✓			CHAOS long form	HC as mediator and moderator in relationships between maternal and paternal ADHD symptoms and parenting
Nelson, 2009 [93]	101		7y	✓			CHAOS long form	Parental supportive and nonsupportive responses to child
Pike, 2016 [94]	106		Siblings aged 4–8 years at baseline & 9–13 years at follow-up		✓		CHAOS short form	Siblings’ perceptions of mothering and fathering
Spilsbury, 2017 [96]	26		11-12y	✓^d^			CHAOS long form	Sleep-disturbing behaviours of family members
Valiente, 2007 [75]	188		7-12y	✓			CHAOS short form	Positive and negative reactions to child’s negative emotions
Vernon-Feagans, 2012 [85]	1123		2, 6, 15, 24, & 36 m		✓	✓	Interview/ observation	Parenting as a mediator in the relationship between HC and language development
Wang, 2013 [97]	160		3-7y	✓			CHAOS short form	HC as a moderator in relationship between maternal attribution bias (e.g. whether they attribute child misbehaviour to the child’s intentions and dismiss situational factors) and parenting behaviour
Whitesell, 2015 [98]	100		1, 3, 6, 9, & 12 m		✓		DISCORD - an observer reported measure of HC	SES; life events; co-parenting; emotional availability at bedtime
Wirth, 2017 [99]	84		7-13y				CHAOS long form	HC as mediator in relationship between children’s ADHD symptoms and inadequate parenting
Zvara, 2014 [100]	962		2, 6, 15, 24, 60 months & 1st grade		✓	✓	Interview/ observation	Children’s representations of family dysfunction from drawings; mediating role of parenting in relationship between HC and family dysfunction
**Household Characteristics and Food Security**								
Fiese, 2016 [90]	221		Elementary school (USA)	✓		✓	CHAOS short form	Food security
Martin-Biggers, 2018 [101]	550		2-5y	✓			CHAOS short form	Family meals; food availability/security; family meal atmosphere
Pinard, 2015 [95]	252		0-18y	✓		✓	CHAOS short form	Food security
**Parent Outcomes** [12, 101–105]								
Deater-Deckard, 2012 [12]	153		33-88 m^e^	✓			CHAOS short form	Maternal EF; SES as moderator of relationship between HC and maternal EF
MacRae, 2017 [102]	44		1 ½ - 5y^e^	✓			CHAOS long form	Parent fat intake and cortisol levels
Madigan, 2017 [103]	501		2-54 m^e^		✓		Adapted version of the HOME scale	Maternal depression trajectories
Martin-Biggers, 2018 [101]	550		2-5y	✓			CHAOS short form	Maternal eating behaviours; weight status; health status
Thomas, 2016 [104]	42		32wks^e^	✓			CHAOS long form	Maternal depression, fatigue, and sleep
Whitesell, 2018 [105]	167		1, 3, 6, 9, 12 m		✓		DISCORD	Parent sleep
**Hormone** [60, 106–112]								
Blair, 2013 [106]	1292		2, 7, 24, & 36 mo, 5y		✓	✓	Interview/ observation	Cortisol and ANS activity
Chen, 2010 [107]	50		9-18y		✓	✓	CHAOS long form	HC as mediator in the relationship between low SES and cortisol profiles
Doom, 2018 [112]	242		35–62, 38–85, 84-122 m		✓	✓	CHAOS long form	Diurnal cortisol levels in response to social stress test
Laurent, 2014 [60]	200		9 m, 18 m, 27 m, 4.5y, 6y		✓		CHAOS short form	Developmental stability of hypothalamic-pituitary-adrenal (HPA) axis activity
Lumeng, 2014 [108]	331		3-4y	✓		✓	CHAOS long form	Morning cortisol levels
Miller, 2013 [109]	34		3-4y	✓		✓	CHAOS long form	Gut biomarkers
O’Brien, 2013 [110]	135		18-66y	✓			CHAOS short form	Stress
Schreier, 2014 [111]	244		13-16y	✓			CHAOS long form	Inflammatory profile
**Physical Health, Health Behaviours & Communication Disorders** [13, 52, 82, 96, 101, 105, 108, 113–124]								
**Health/Disease/Disorder Outcomes**								
Appelhans, 2014 [113]	103		6-13y	✓		✓	CHAOS long form	HC as mediator in relationship between sleep duration and child weight
Chae, 2016 [114]	125		Mean 12.6y	✓			CHAOS long form	HbA1c
Dush, 2013 [115]	3288		3y, 5y		✓	✓	Interview/ observation	Child health
Khatiwada, 2018 [118]	401		Infants; followed during first 12 mo		✓		CHAOS long form	Weight status
Kraft, 2014 [117]	69		Mean 3.7y	✓			CHAOS long form	Stuttering severity
Levin, 2013 [116]	104		1-13y		✓		CHAOS long form	HbA1c
Lumeng, 2014 [108]	331		3-4y	✓		✓	CHAOS long form	Overweight
Martin-Biggers, 2018 [101]	550		2-5y	✓			CHAOS short form	Health status; weight status
Tucker, 2018 [82]	238		Followed between 10th grade (mean at baseline 15.59y) and 12th grade		✓	✓	CHAOS short form	Physical health symptoms; HC as moderator in relationship between hostile parenting and physical health symptoms
**Diet & Dietary Behaviours**								
Asta, 2016 [119]	209		21, 27, & 33 m		✓	✓	CHAOS (not specified)	Eating in the absence of hunger
Goulding, 2015 [120]	287		4-8y	✓		✓	CHAOS long form	Maternal feeding goals
Martin-Biggers, 2018 [101]	550		2-5y	✓			CHAOS short form	Food-related behaviours
**Sleep**								
Bartel, 2016 [121]	1402		12-19y	✓			CHAOS short form	Sleep
Billows, 2009 [122]	217		13-18y	✓			CHAOS long form	Sleep
Boles, 2017 [13]	72		Mean 4.7y	✓		✓	CHAOS long form	Sleep; HC as a mediator in relationship between behaviour and sleep outcomes.
Gregory, 2005 [52]	6612		3y, 4y		✓		CHAOS short form	Sleep; HC as mediator relationship between sleep problems and anxiety
Spilsbury, 2017 [96]	26		11-12y	✓^d^			CHAOS long form	Sleep
Whitesell, 2018 [105]	167		1, 3, 6, 9, 12 m		✓		DISCORD	Sleep
**Other Health Behaviours/Outcomes**								
Brown, 2010 [123]	70		4–7.5y	✓		✓	CHAOS long form	TV viewing behaviours in a laboratory setting
Pesch, 2015 [124]	278		4-8y	✓		✓	CHAOS long form	Maternal perceptions about physical activity.

*C/O* Cross-sectional/other, *L* Longitudinal, *HC* Household Chaos, *EF* Executive function, *CP* Conduct problems, *CU* Callous-unemotional traits, *ANS* Autonomic nervous system

^a^Age for cross-sectional/other study type presented as range (preferential; based on inclusion criteria), mean (if range not given), or age group (if specific age not provided; when age group is used the country where the study took place is also included, as definitions of age groups differ between countries); for longitudinal studies age at each follow-up was provided where available for range, mean, or age group

^b^If multiple selected, then cross-sectional and longitudinal analyses conducted. CHAOS short- and long-form refer to the short-form [23] and long-form [22] versions of the Confusion, Hubbub, and Order Scale (CHAOS)

^c^Temperament-parenting-interactions = interactions between child temperament and parenting (i.e. maternal negativity and positivity)

^d^Reported as longitudinal but conducted as a 14-week laboratory sleep study

^e^Age of the child

#### Cognitive and academic

Sixteen manuscripts were identified that investigated the relationship between household chaos and cognitive/academic outcomes [3, 7, 11, 24–36]. The studies covered the spectrum of age groups, from very early childhood to later adolescence, although the majority were conducted in young children (i.e. ≤5 years). Outcomes assessed included executive function, IQ, general cognitive ability, and a range of academic measures, including reading comprehension, academic achievement, study skills, and learning. Overall, household chaos was consistently associated with adverse cognitive and academic outcomes. There was also evidence for an effect of household chaos on outcomes after controlling for SES [3, 30, 32]. However, null findings were reported in 2 of the 16 studies; one study (*n* = 203) did not find a significant relationship between household chaos and cognitive performance [33], while another study (*n* = 65) failed to demonstrate a significant relationship between household chaos and intelligence, academic achievement, and executive functioning in 6–16 years olds [36].

#### Socio-emotional and behavioural

The majority of studies investigated the relationship between household chaos and socio-emotional and behavioural outcomes [3, 4, 6, 10, 14, 24, 25, 28, 29, 31–33, 37–83], although 18/59 of these studies only investigated household chaos as a mediator or moderator in the relationship between a predictor and a socio-emotional/behaviour outcome. Outcomes included responses to challenges, social skills/competence, emotion regulation, risky behaviours, attention, aspirations, aggression, conduct problems, and callous-unemotional traits. Overwhelmingly, household chaos was shown to be associated with adverse outcomes in both younger children and adolescents [3, 4, 6, 10, 25, 31–33, 38, 41–44, 49, 55, 57, 62–64, 67, 68, 72, 80, 82, 83]; however, it was not shown to be associated with self-regulation and effortful control [53, 83], empathy [64], sexual risk or other violent behaviours in adolescents [44], or occupational aspirations in 7 year olds [47].

#### Communication

A total of six manuscripts investigated the link between household chaos and communication, all of which were conducted in the early childhood setting [6, 7, 23, 25, 84, 85]. Outcomes assessed included non-verbal abilities, receptive and expressive language, and phonological awareness. Household chaos was consistently linked with adverse effects on communication outcomes across all six analyses. After controlling for all other measures of household chaos, lack of routines was significantly associated with lower receptive vocabulary scores in 5 year olds [6], and in an analysis that controlled for 13 covariates, including maternal education and poverty, household disorganisation was associated with significant decreases in both receptive and expressive language in 3-year-old children (*n* = 1145) [85]. Finally, when investigating the heritability of cognitive abilities as a function of the child’s early environment, household chaos, which is classified as a proximal environmental determinant, had stronger effects than distal environmental determinants (e.g. SES) on the heritability of verbal ability [84].

#### Parenting, and family and household functioning

Twenty-one studies investigated the effects of household chaos on parenting and family functioning [4, 10, 45, 75, 85–101], of which five focused on the role of household chaos as a mediator or moderator. Outcomes assessed included parenting, parent-child interactions, discipline, sibling relationships, parental response and reactions to child behaviours, family dysfunction, and food insecurity. Household chaos was associated with increased parent-child conflict, decreased parent-child closeness, decreased supportive parenting, decreased positive parenting, and increased negative parenting [87], in addition to less favourable co-parenting, and less emotional availability at bedtime [98]. A chaotic home environment was also shown to be associated with less responsive and less stimulating parenting [88], less effective parental discipline [10], greater non-supportive responses to children’s emotions and fewer supportive responses [75, 93], and greater paternal hostility [94]. Greater chaos in the home was associated with increased odds of household members disturbing the efforts of adolescents to fall asleep, and decreased the odds of adolescents reporting that nothing was keeping them awake or making it difficult to sleep [96]. Finally, cumulative family disorganisation, but not cumulative family instability, was indirectly associated with children’s representation of family dysfunction in drawings, through parenting behaviours [100].

Food insecure households were more likely to have greater household chaos scores compared with food secure households, even after controlling for education and marital status, with higher chaos homes reporting less planning around mealtimes [90]. In another study, high and medium household chaos homes were more likely to experience low or very low food security compared with low chaos homes [95]. Further, low chaos predicted greater availability of fruits and vegetables in the home and more family meals, while high chaos was a significant predictor of food insecurity risk and greater availability of salty and fatty snacks [101].

#### Parent outcomes

The relationship between household chaos and parent outcomes, including maternal executive function, parent sleep, parent feeding behaviours and weight status, and maternal depression, were assessed in six studies [12, 101–105], of which two studies included sample sizes of less than 50 participants [102, 104], and all of which were conducted in the early childhood setting. Household chaos was associated with poorer maternal executive function (*n* = 153), although the modest effect of household chaos overlapped with the effects of co-varying factors, including SES and verbal ability [12]. In parents of children aged 18 months to 5 years (*n* = 44), household chaos was significantly associated with fat intake and high serum cortisol levels; however, the relationship between household chaos and fat intake appeared to be somewhat mediated by cortisol levels, although this relationship was not significant [102]. In mothers of children aged 2–5 years (*n* = 550), high chaos was associated with greater engagement in emotional and disinhibited eating, while mothers in low chaos households were more likely to be adventurous eaters [101].

Household chaos was lower in mothers with trajectories of low-stable levels of depression compared with moderate-increasing levels of depression, but not in those with remitting depression [103]. In mothers of infants [104], household chaos was strongly correlated with measures of maternal depression, sleep, wake disturbances, and fatigue. Similarly, mothers and fathers of infants in high chaos homes demonstrated greater variability in sleep duration compared with low chaos families, while parental sleep fragmentation mirrored that of the child in low chaos homes, where fragmented sleep decreased for both the parents and child over the course of the first year [105].

#### Hormones

Eight studies investigated the role of household chaos in cortisol and autonomic nervous system activity, inflammatory profiles, and gut biomarkers [60, 106–112], with sample sizes ranging from 32 to 1292 participants. For stress physiology, household chaos in early childhood was associated with (1) a blunted diurnal cortisol slope in middle school [112], (2) cortisol levels in 7 year olds who had lower levels of resting heart rate variability [106], (3) stable morning cortisol levels in 6 year olds [60], and (4) lowered morning cortisol levels in 3–4 year olds [108]. In 13–16 year olds [111], household chaos was associated with increased systemic inflammation, interleukin-6 (IL-6) levels, and C-reactive protein levels, although the relationship between chaos and systematic inflammation and IL-6 levels was moderated by SES. In participants aged 18–66 years, household chaos was not related with hair cortisol levels [110] or gut-derived biomarkers associated with appetite and regulation [109].

#### Physical health, health behaviours and communication disorders

Overall, 19 analyses were conducted that investigated the role of household chaos on health and health behaviours: (1) 9 papers specifically looked at physical health outcomes, disease, and communication disorder outcomes [82, 101, 108, 113–118], including glycaemic control, child health, weight status, and stutter, (2) 3 studies investigated diet and dietary behaviours [101, 119, 120], (3) 6 studies looked at sleep [13, 52, 96, 105, 121, 122], and (4) 2 studies assessed other outcomes, including TV viewing behaviours in a laboratory setting [123] and mothers’ perceptions on children’s physical activity [124]. For glycaemic control, both maternal and paternal household chaos scores were positively associated with HbA1c in children and adolescents with type 1 diabetes [114] and in children aged 1–13 years with type 1 diabetes mellitus [116]. Chaotic homes were also linked with low cortisol levels (hypocorticolism) in children aged 3–4 years, which in turn predicted overweight in girls, both directly and indirectly through the mediating role of satiety responsiveness, and in boys, indirectly through the mediating role of emotional overeating [108]. In infants followed during the first year of life, household chaos was also found to significantly predict weight-for-height z-scores, even after controlling for possible confounders [118].

Chaos was found to be associated with maternal feeding goals [120], with lower household chaos associated with more positive maternal feeding goals, such as promotion of child autonomy around eating. Greater household chaos was also shown to be associated with greater consumption of sugar-sweetened beverages in preschoolers [101]. Further, chaos was associated with mother-report of general child health, even after controlling for SES, maternal health status, and family structure [115]. Similarly for older children, self-report of household chaos in 10-year-olds was associated with worse physical health 2 years later [82].

With respect to sleep, a more chaotic home environment was associated with mixed effects on sleep outcomes in adolescents, including sleep onset latency and sleep duration [121, 122]; however, in younger children greater scores for household chaos were significantly associated with higher parent-reported scores for bedtime resistance, sleep anxiety, and total sleep problems [13]. One study found that infants from highly chaotic homes demonstrated delays in sleep consolidation patterns and greater fragmentation of sleep; however, they also reported longer and more variable sleep duration compared with infants in low chaos homes [105]. The authors suggested that this unexpected difference in sleep duration may have reflected a higher quality of sleep in the low chaos households, where sleep was less fragmented and bedtimes and wake times less variable. Chaos in the home environment was not found to be associated with maternal perceptions about physical activity in children [124], stutter severity in children with stutter [117],TV viewing behaviours (i.e. looking patterns) in a laboratory setting [123], or eating in the absence of hunger in low-income toddlers [119].

#### Mediation and moderation analyses

Table 5 presents results from studies where mediation and moderation analyses were conducted. Household chaos was consistently found to mediate the relationship between predictors of adverse child outcomes. Importantly, a number of studies demonstrated the mediating role of chaotic homes between SES and outcomes [14, 26, 107], suggesting that the adverse effects of low SES on child outcomes may, at least in part, be mediated by the effects of household chaos. Further, chaotic environments were also shown to moderate the relationship between several predictors of adverse children and family outcomes, including SES [50], parenting [24, 45, 58], and parental executive function [40], whereby the effects of these predictors were more pronounced in highly chaotic homes. A number of studies also demonstrated the mediating role of parenting on the relationship between household chaos and adverse child outcomes (Table 6) [6, 76, 85, 91], where adverse parenting behaviours may partially explain the relationship between chaos and child outcomes.

**Table 5:** Household Chaos as mediator and/or moderator

Study	HC as mediator	HC as moderator
Asbury, 2003 [37]	HC did not mediate the relationship between parenting and behavioural outcomes.	HC significantly moderated the relationship between (1) differential harsh parental discipline and differential CP, and (2) differential negative parental feelings and anxiety, hyperactivity, and CP. That is, the relationship between NSE influences (i.e. differential parenting) and behavioural outcomes were stronger in high chaos homes.
Asbury, 2006 [24]		HC moderated the relationship between: (1) discordant harsh discipline and CP [increase in CP], (2) discordant negative feelings and CP [increase in CP], (3) discordant parental negative feelings and academic achievement [achievement decreased], and (4) instructive parent-child communication and anxiety [anxiety increased] (where instructive communication including things like correcting child’s grammar)
Boles, 2017 [13]	Children with higher scores for emotional and behaviour problems were more likely to have higher bedtime resistance, but higher levels of HC may account for this relationship; HC associated with bedtime resistance significantly mediated the relationship between Behavioural and Emotional Screen System and bedtime resistance.	
Bridgett, 2013 [39]	HC mediated the effects of maternal self-regulation on infant frustration/distress to limitations.	
Brieant, 2017 [40]		Parent EF significantly predicted changes over time in adolescent EF, with this association contingent on levels HC, such that a stronger relationship was observed in the presence of greater HC.
Chen, 2010 [107]	HC partially mediated the relationship between SES and daily cortisol output.	
Chen, 2014 [45]		HC moderated the relationship between harsher maternal negativity and child maladjustment in children who had low levels of effortful control. HC did not moderate any other temperament-by-parenting interactions.
Coldwell, 2006 [4]		In a minority of cases, HC was found to moderate the role between parenting and child behaviour, whereby HC exacerbated the effect of poorer quality parenting on problem behaviour.
Deater-Deckard, 2012 [89]		Maternal EF moderated the relationship between child CP and harsh parenting, so that this relationship was only significant in mothers with poorer EF. Further, this moderating effect of EF was further moderated by HC, so that the effect was particularly strong in the presence of low HC, but not high HC.
Evans, 2005 [14]	The adverse effects of poverty on socioemotional adjustment were shown to be mediated by HC.	
Farbiash, 2014 [46]	HC was found to significantly mediate the relationship between paternal (but not maternal) ADHD symptoms and children’s aggression in the preschool years.	
Flouri, 2017 [47]	HC did not mediate the relationship between low SES and CP in children with ADHD	
Fuller-Rowell, 2015 [50]		Early HC moderated relationship between poverty and task persistence in later adolescence.
Garrett-Peters, 2016 [26]	In the presence of household disorganisation (but not instability), income poverty was no longer directly related to academic achievement. Income was related to disorganisation, however, which in turn was associated with lower academic achievement.	
Gould, 2017 [51]		HC did not moderate the relationship between genes and development of ADHD.
Gregory, 2005 [52]	HC partially mediated the relationship between sleep problems and child anxiety, with HC accounting for ~ 30% of the association.	
Hart, 2007 [11]	HC mediated the relationship between shared environmental variance and longitudinal stability of cognitive ability in early childhood.	
Hur, 2015 [28]	HC mediated relationship between parental depressive symptoms and mother-reported social skills of child.	
Kahn, 2016 [58]		Hostile parenting mediated the relationship between parent and adolescent CU, but only in the presence of high HC. Harsh parenting explained intergenerational similarity in CU traits, and HC exacerbated this relationship.
Kim-Spoon, 2017 [59]		High parental control predicted better neural cognitive control in adolescents living in low HC contexts. Poor neural cognitive control was associated with reduced social competence 1-year later (after controlling for social competence at baseline), but this relationship was only significant in high HC contexts. HC was shown to undermine positive associations between parental control and adolescent neural cognitive control and exacerbated the negative association between poor neural cognitive control and social competence development.
Lauharatanahirun, 2018 [81]		HC moderated the relationship between adolescent-report of parental knowledge/parental monitoring and adolescent insular risk processing, which has been shown to precede risk-adverse choices, whereby it was only present in the presence of low HC.
Lemery-Chalfant, 2013 [61]		Heritability of children’s temperament was moderated by HC, meaning that effortful control and surgency were more heritable in high HC contexts.
Midouhas, 2013 [62]		Autism was associated with higher rates of psychopathology over time, and family poverty was associated with emotional and CP (psychopathology); however, home organisation did not moderate the relationship between family poverty and psychopathology.
Mills-Koonce, 2016 [64]		HC did not moderate the relationship between parenting and child behaviours.
Mokrova, 2010 [92]	Mother’s ADHD symptoms were positively associated with inconsistent discipline and non-supportive responses to child’s negative emotions; this relationship was mediated by HC. Father’s ADHD symptoms were associated with low involvement; HC mediated this relationship.	Father’s ADHD symptoms were associated with inconsistent discipline; this relationship was moderated by HC.
Oliver, 2008 [65]		HC moderated the relationship between within-pair differences in perceptions of the classroom environment and teacher report of less prosocial and more hyperactive behaviour, and greater conduct and peer problems. Specifically, associations between differential classroom environment and CP were greater for children in more chaotic homes.
Petrill, 2004 [23]	HC partially mediated an independent and significant portion of the shared environment for verbal and nonverbal and PARCA scores at ages 3 and 4 years. HC was a significant mediator even when controlling for SES.	
Shelleby, 2014 [70]	Significant direct association between income and emotional problems and CP; HC was a significant mediator in the relationship between income and emotional problems.	
Taylor, 2014 [35]	HC mediated relationship between respect for rules and increased reading comprehension, even after controlling for income.	
Towe-Goodman, 2011 [73]		In a low-income sub-group, HC was found to moderate the relationship between interparental aggression in toddlerhood and the development of child attention skills and early childhood behaviour problems at age 3 years. In low-income families with low HC, there was a significant association between greater interparental aggression and increases 3-year ADHD symptoms, but this relationship was not significant in low-income, high HC families.
Tucker, 2018 [82]		HC moderated the effect of hostile parenting on adolescents’ depression 2 years later, so that depression was exacerbated in the context of high HC. HC did not moderate the relationship between parenting and physical health and problematic substance use.
Wang, 2012 [78]		HC moderated the genetic variance and covariance between externalising problems and attention regulation, with the genetic influences stronger in HC contexts; however, higher levels of HC attenuated the genetic association between externalising problems and attention regulation.
Wang, 2013 [97]		The relationship between maternal attribution bias and parenting behaviour were stronger in more chaotic environments; the moderating effect of HC was particularly strong for internal attribution bias.
Wilkinson, 2013 [79]		Depressive symptoms at age 12 were significantly heritable, and HC and parenting style at age 9 years moderated this association.
Wirth, 2017 [99]	The relationships between ADHD and positive parenting, corporal punishment, and inconsistent discipline were somewhat mediated by HC. As such, high HC was associated with specific parenting dimensions in families with children with ADHD.	

*NSE* Non-shared environment

**Table 6:** Mediation and moderation analysis of relationship between household chaos and outcome

Study	Relationship between HC and outcome mediated	Relationship between HC and outcome moderated
Asbury, 2005 [84]		There was greater group heritability for verbal ability, but not non-verbal ability, in early childhood within the context of higher HC.
Berry, 2016 [25]		Relationship between increased household disorganisation and decreased cognitive and social outcomes was somewhat moderated by childcare hours (i.e. greater childcare hours attenuated the relationship)
Brown, 2008 [42]	Sleep problems partially mediated the relationship between HC and hopeless/helpless responses to academic challenge.	
Corapci, 2002 [88]	Overall neither parental mood nor self-efficacy was found to mediate the relationship between HC and parenting.	
Deater-Deckard, 2012 [12]		The relationship between HC and maternal EF was moderated by SES, meaning the adverse effects of HC on maternal EF may be particularly important within socioeconomically distressed contexts.
Fisher, 2018 [80]		The quality of classroom climate across 3 years of elementary school was not found to moderate the relationship between HC and adolescent outcomes.
Khatiwada, 2018 [118]	HC was positively associated with weight status at 12 months of age; however, this relationship was not mediated by breastfeeding, sleep, or screen time.	
Kretschmer, 2009 [91]	The relationship between HC and sibling relationship quality was mediated by parenting, including maternal warmth and paternal harsh discipline.	The relationship between HC and sibling relationship quality was moderated by maternal harsh discipline.
Lemery-Chalfant, 2013 [61]	Lower HC was associated with higher effortful control in children, and this association was genetically mediated.	
Martin, 2012 [6]	Lack of routines was associated with lower receptive vocab and delayed gratification. In homes with the TV usually on, children had greater aggression scores and attention problems. The association between routines and receptive vocab was partially mediated by provision of learning materials. The association between lack of routines and delayed gratification was not mediated by maternal warmth or provision of learning materials, suggesting routines in and of themselves were associated with development of early self-regulation.	
Miller, 2017 [63]		HC was negatively associated with emotion regulation, but this relationship was not moderated by cortisol levels. The relationship between routines and emotion regulation was moderated by cortisol level, meaning that lack of routines was more strongly associated with poor emotion regulation in children with lower cortisol output.
Mills-Koonce, 2016 [64]	Maternal sensitivity and harsh parenting mediated the relationship between HC and child behaviours.	
Supplee, 2007 [71]	Maternal report of HC was associated with children’s externalising behaviour at age 4 years, even after controlling for SES and ethnicity.	HC was positively associated with teacher report of externalising problems at school at age 5.5 years. Maternal monitoring was not found to moderate the effects of HC on externalising behaviour.
Tucker, 2015 [74]		HC predicted less positive adolescent beliefs about mastery, future obstacles, and having a successful career; mother’s hostility moderated the relationship between HC and future obstacles and stress about the transition to emerging adulthood.
Vernon-Feagans, 2012 [85]	Household disorganisation was associated with variance in receptive and expressive language; parenting partially mediated this relationship.	
Vernon-Feagans, 2016 [76]	Disorganisation in the home was indirectly associated with behavioural regulation through intermediate impacts on parenting behaviours and children’s early executive function skills.	
Zvara, 2014 [100]	Cumulative family disorganisation, but not cumulative family instability, was found to have a significant indirect on children’s representation of family dysfunction through parenting behaviours. As such, the proximal effects of daily disorganisation appeared to outweigh the effects of periodic instability overtime.	

## Discussion

The aim of this scoping review was to provide a general summary of the extant research investigating household chaos in children and their families, with respect to (1) measurement tools used, (2) study details, and (3) outcomes assessed. The review found that research to date predominantly relied on either the short- or long-form version of the CHAOS scale, was conducted in either the U.S or U.K, and utilised primarily cross-sectional or longitudinal study designs. Almost a quarter of the research was undertaken in large, nationally representative samples of over 1000 study participants, and mostly focussed on young, healthy children. Both direct and indirect relationships between household chaos and outcomes were investigated, with the majority of studies assessing the relationship between household chaos and socio-emotional and behavioural outcomes. While an in-depth analysis of findings and assessment of bias was beyond the scope of this review, a preliminary summation of the research showed consistency in the evidence for significant relationships between household chaos and adverse effects across seven categories of outcomes in diverse populations with respect to age, disease status, and SES. To the best of our knowledge, this scoping review is the first review to map the literature within this field.

The review demonstrated that household chaos appears to influence a range of child, parent, and family outcomes. The breadth of these findings are not surprising given the diverse ways household chaos potentially interferes with processes in the home environment known to support healthy child development [125]. For example, evidence suggests that parenting behaviours and parent-child interactions are compromised within chaotic home environments [12, 39, 89, 126]. Parents in chaotic homes have been shown to be less responsive, less stimulating, more likely to interfere with children’s attempts at exploration, less likely to provide scaffolding, and more likely to engage in harsher discipline [2]. Not surprisingly, parenting was found to mediate the relationship between household chaos and a number of outcomes, including sibling relationship quality [91], child behaviours [64], receptive and expressive language [85], and children’s representation of family dysfunction [100]. Yet, it was also shown that even in the presence of positive parenting, such as maternal monitoring, the adverse effects of household chaos remain, suggesting that the negative effects of living in a chaotic home environment may not always be overcome by positive parenting practices [71].

It has also been suggested that the mechanism through which chaos impacts on parents is through increasing levels of stress and distraction, resulting in reduced regulatory functioning of the prefrontal lobe, and thus rendering even parents with normal to high emotional regulation and cognitive control compromised in their ability to parent effectively [127]. For example, household chaos has been shown to moderate the relationship between parenting behaviours and child outcomes, whereby chaos exacerbates the effects of negative parenting behaviours and undermines the effects of positive parenting behaviours [4, 24, 26, 58, 73, 92, 97].

Household chaos has also consistently been found to impact on measures of stress physiology in young children, and thus may represent a form of toxic, albeit low-level, stress [106, 128]. Toxic stress in childhood has been linked with increased risk of negative health outcomes in later life [129], and one potential pathway connecting toxic stress in childhood and adult health is through an altered stress response [130]. Exposure to household chaos during the preschool years was shown to be associated with a blunted diurnal cortisol slope in middle childhood [112], and further, household chaos was found to partially mediate the relationship between low SES and cortisol levels [107]. These findings are concerning as a blunted diurnal cortisol slope, which is considered maladaptive, has been identified as a precursor to a number of diseases and disorders in adulthood [131]. As such, household chaos may signify an aspect of toxic stress in childhood that should be considered by public health researchers.

It may also be that household chaos impedes child development directly through effects on attention allocation and information-processing skills [1]. In the context of a home environment with high frequency or high levels of distractions and background noise, the child develops adaptive techniques for filtering out stimulation; however, these techniques may not be selective, meaning that stimulation that facilitates development is also inadvertently filtered out [132]. Importantly, technological advances in the last 10 years may have created greater opportunities for children to be exposed to background media stimulation. For emphasis, it is possible that newer media devices, such as Smartphones, have increased the level of background media distraction that children are exposed to in the modern home environment. These newer devices not only interfere with child attention processes, but can also reduce responsive parenting behaviours. A recent review found that increased mobile connectivity distracts parents from parent-child interactions, and that distracted parents are more likely to be less responsive and sensitive to the needs of their children [133]. Further, a 2018 cross-sectional study in preschool-aged children (*n* = 385) found that greater levels of household chaos were associated with increased total screen use in pre-schoolers and screen-use behaviours related to disrupted nighttime sleep [134]. Future research will need to determine whether screen use mediates the relationship between household chaos and outcomes, or alternatively, whether new media devices contribute to the household chaos construct itself.

We also identified a number of studies demonstrating the mediating role of household chaos between risk factors and adverse child outcomes. Of particular interest was the potential role household chaos plays in explaining, at least in part, the relationship between SES and chid outcomes. For example, household chaos was found to mediate the relationship between low SES and daily cortisol output [107], socioemotional adjustment [14], academic achievement (via the disorganisation pathway rather than the instability pathway) [26], and emotional problems [70], but not conduct problems in children with ADHD [47]. More generally, household chaos was also shown to mediate relationships between child sleep and anxiety [52], child behavioural problems and sleep resistance [13], and maternal self-regulation and infant distress to limitations [39].

Finally, household chaos was also shown to moderate the relationship between risk/protective factors and outcomes. For example, children in high chaos homes were shown to be more vulnerable to risk factors, such as hostile parenting [82], and less likely to benefit from positive parenting practices, such as parental control [59]. Alternatively, children in lower chaos homes appeared more likely to benefit from protective factors, such as parental monitoring [81]. A systematic review of these studies is required to document the evidence supporting the mediating and moderating roles of household chaos between risk/protective factors and child outcomes.

This review had a number of strengths and limitations. The scoping review methodology allowed us to map a heterogeneous research area, providing an overview of research within a field that has previously escaped comprehensive review [19]. It also enabled us to summarise research using a variety of study designs and methodologies, and assess a large number of outcomes across several categories. As a result, this scoping review provides a comprehensive overview of published evidence investigating the construct of household chaos, with no limitations on study design, outcomes of interest, context, or age groups. Further, the review appears timely, given the potential for an increasing level of chaos in the daily lives of families [115], in addition to the increasing number of studies published in the field in recent years.

However, scoping reviews are not without their limitations. While we provided a general narrative overview of findings, more work is required to further analyse and synthesise the findings reported in the included studies. Additionally, we did not conduct an assessment of study quality, in line with scoping review guidelines [15], and therefore it is not possible to comment on the quality of the research reported herein, and further, while we followed recommended guidelines for conducting scoping reviews, we did not undertake the optional consultation process. The considerable heterogeneity across studies, with respect to study designs, methodologies, and outcomes of interest, may also be considered a weakness, as it did not allow in-depth synthesis of the findings, and therefore difficulty in identifying nuances in the research, such as critical windows of exposure. Finally, our reliance on published data only subjected the review to publication bias; however, the decision was made to exclude the grey literature, given the large number of published studies identified by the original search.

Our findings highlighted that the majority of research in the area has been conducted in U.S and U.K populations, and as such, studies from other countries may be needed to better understand how chaotic home environments affect families within different cultural contexts. Research is also needed to assess whether household chaos has been increasing in recent years, and if so, if this increase is across the population or only within specific sub-groups. Drivers of this potential change should also be investigated. For example, widespread social changes may have reduced a family’s ability to engage in routines, an important pathway by which household chaos may negatively affect children [6]. Families today are more likely to have two parents who work [135], and children who are cared for outside the family home [136]. These activities may increase family disorganisation and environmental confusion, through increasing logistical demands, feelings of hurriedness, and increasing perceptions of time scarcity, which in turn may result in decreased engagement in important family routines and rituals [137]. Finally, while high levels of household chaos negatively influence children and families, it is unknown whether there is an ideal lower limit of household chaos, below which adverse effects of an ‘overly structured household’ may occur.

Identifying, targeting, and effectively reducing household chaos may offer a unique course of action for (1) improving child, parent, and family outcomes, (2) tackling social, behavioural, cognitive, and health problems linked with low SES in childhood [107], and (3) increasing the impact of family-based programmes designed to improve child outcomes. Yet we do not believe it is possible to make recommendations for practice as our scoping review did not assess the methodological quality of the included research [15]. Before recommendations can be made, we suggest the need for systematic reviews that focus on specific outcomes of interest, pathways linking chaos and outcomes, and the mediating and moderating role of household chaos between risk and protective factors and child outcomes. These reviews would allow for an assessment of study quality and would enable recommendations to be made about how findings could inform practice. Studies are also needed to assess whether it is possible to reduce household chaos. To the best of our knowledge, no interventions have been undertaken with the primary aim of targeting household chaos and as such no tools are currently available that have demonstrable effectiveness in reducing chaos in the family home. Further, it remains to be seen whether a reduction in household chaos actually translates into positive outcomes for children, parents, and families.

## Conclusions

Our review identified a diverse body of literature investigating the construct of household chaos. We found that chaotic home environments appear to correlate with a broad spectrum of adverse child, parent, and family outcomes, potentially describe, at least to some extent, the relationship between low SES and adverse outcomes, undermine positive parenting behaviours, and exacerbate negative parenting behaviours. Future research is needed to investigate whether household chaos has been increasing in recent years, what factors may have driven the hypothesised increase in household chaos, whether newer mobile media devices create greater opportunity for the experience of household chaos, and, if so, how to capture this in a new or updated measurement tool. The effects of the construct on outcomes also need to be investigated in other cultural contexts, and programmes developed to not only investigate how household chaos can effectively be reduced, but also assess whether a reduction in household chaos translates into improved outcomes. Before recommendations to inform practice can be made, we propose the undertaking of systematic reviews looking at specific outcomes of interest and the pathways through which household chaos impacts on child, parent, and family outcomes.

## Data Availability

Not applicable.

## Abbreviations

TV: Television
SES: Socio-economic status
CHAOS: Confusion, Hubbub, and Order Scale
